# Macromolecular crowding directs the motion of small molecules inside cells

**DOI:** 10.1098/rsif.2017.0047

**Published:** 2017-06-14

**Authors:** Stephen Smith, Claudia Cianci, Ramon Grima

**Affiliations:** School of Biological Sciences, University of Edinburgh, Mayfield Road, Edinburgh EH9 3JR, UK

**Keywords:** macromolecular crowding, volume exclusion, Brownian dynamics, advection–diffusion equations

## Abstract

It is now well established that cell interiors are significantly crowded by macromolecules, which impede diffusion and enhance binding rates. However, it is not fully appreciated that levels of crowding are heterogeneous, and can vary substantially between subcellular regions. In this article, starting from a microscopic model, we derive coupled nonlinear partial differential equations for the concentrations of two populations of large and small spherical particles with steric volume exclusion. By performing an expansion in the ratio of the particle sizes, we find that the diffusion of a small particle in the presence of large particles obeys an advection–diffusion equation, with a reduced diffusion coefficient and a velocity directed towards less crowded regions. The interplay between advection and diffusion leads to behaviour that differs significantly from Brownian diffusion. We show that biologically plausible distributions of macromolecules can lead to highly non-Gaussian probability densities for the small particle position, including asymmetrical and multimodal densities. We confirm all our results using hard-sphere Brownian dynamics simulations.

## 1. Introduction

Cells are highly crowded environments, with up to 40% of the cytoplasmic volume occupied by macromolecules such as RNA, ribosomes and enzymes [1,2]. The motion of smaller molecules, such as amino acids and small proteins, is seriously impeded by macromolecular crowding: a large number of *in vitro* studies have shown that diffusion coefficients are reduced and binding rates increased in the presence of synthetic obstacles like dextran and Ficoll [2–7]. Furthermore, modern fluorescence microscopy techniques allow direct observation of single-particle motion *in vivo*, and experiments have shown that biomolecules diffuse in an anomalous manner, in particular, subdiffusively [8–10] and superdiffusively [11]. Theoretical approaches to crowding are generally simulation-based: particularly popular are highly detailed Brownian dynamics (BD) models [12–14], and cruder lattice-based descriptions [15–19].

However, nearly all *in vitro* and theoretical treatments of crowding consistently overlook the fact that the cell is not a homogeneous environment. Even in prokaryotes, where the cell interior is completely membrane free, distinct sub-cellular compartments exist. Firstly, there is a clear demarcation between the cytoplasm and the nucleoid owing to a significant difference in the concentration of macromolecules [20,21]. Secondly, macromolecules are actively transported to opposite ends of the cell in preparation for cell division, leading to a bimodal crowder distribution [22,23]. Thirdly, phase separation is known to occur in the cytoplasm owing to hydrophobic and elecrostatic interactions between different macromolecular species, leading to distinct regions of high and low crowder density [24]. These effects imply that the cell interior consists of a highly non-uniform distribution of crowders which is maintained over long time scales.

In this article, we address the question of how a purely steric heterogeneous crowder distribution affects the motion of a small particle. Though it is frequently claimed that a purely steric model of crowding cannot account for the full variety of behaviours observed *in vivo* [25,26], we show here that a steric description can explain a considerably wider variety of phenomena than usually thought, including multimodal densities, directed motion, and super- and subdiffusion. This highly irregular behaviour is caused directly by the heterogeneity of the crowded environment, and so naturally would not be apparent in *in vitro* or computational studies which assume uniform crowder distributions.

In §2 we derive, from a microscopic description, a pair of diffusion equations for a population of large particles and a single small particle. We perform a perturbative expansion in the ratio of particle sizes, and thereby obtain a single advection–diffusion equation for the small particle motion, which depends strongly on the spatial distribution of the large particles. In §3, we investigate how a variety of biologically plausible distributions of macromolecules might affect the motion of a small particle. We confirm the predictions of our advection–diffusion equation with hard-sphere BD simulations. We conclude with a discussion in §4.

## 2. Diffusion equations with macromolecular crowding

**Figure 1.**
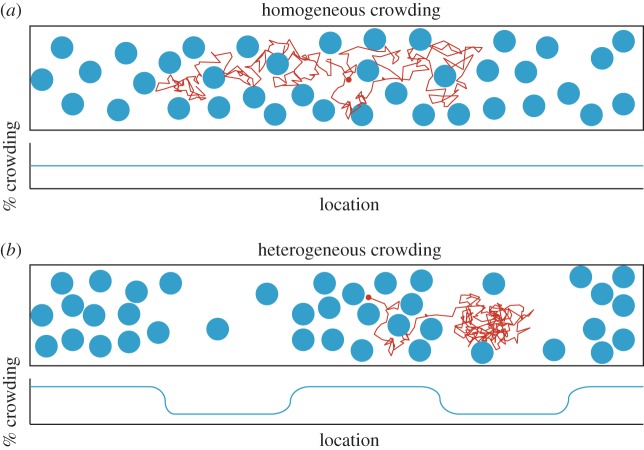
Cartoons showing the difference between homogeneous and heterogeneous crowding. (*a*) A uniform distribution of large particles (blue) corresponds to homogeneous crowding. A small particle (red) will tend to exhibit Brownian diffusion, with a reduced diffusion coefficient. (*b*) A non-uniform distribution of large particles (blue) corresponds to heterogeneous crowding. A small particle (red) will tend to be directed towards less-crowded regions.

Mathematical models of macromolecular crowding tend to assume that macromolecules are homogeneously (uniformly) distributed throughout the cell, but in reality the local concentration of macromolecules can vary widely on a subcellular length scale (see Introduction). The consequences of this discrepancy are demonstrated in figure 1. The top cartoon shows a typical trajectory of a small Brownian particle (red) in a homogeneous distribution of macromolecules (blue) at a moderate level of crowding. The trajectory, starting in the centre of the volume (red circle), is essentially Brownian, although frequent collisions with macromolecules will tend to reduce the small particle's diffusion coefficient. The bottom cartoon shows a typical trajectory of a small Brownian particle (red) in a heterogeneous distribution of macromolecules (blue), with alternating regions of high and low crowding. In this case the small particle, again starting from the centre (red circle), is directed preferentially towards a region of low crowding, and—since it is then trapped between regions of high crowding—it will tend to remain there much longer than is predicted by a standard diffusion equation. Although both cases in figure 1 have the same overall level of crowding, the behaviour of a small particle varies greatly between the two. In this section, we therefore attempt to derive a diffusion equation for the small particle which can capture the irregular motion induced by heterogeneous macromolecular crowding.

We consider the three-dimensional space (−∞, ∞) × [0,*L*] × [0, *L*] with reflective boundaries, in which particles can diffuse in all dimensions, but we are only interested in the first dimension. We consider two species of spherical particles, *X*_1_ and *X*_2_, with radii *r*_1_ and *r*_2_, respectively, and intrinsic diffusion coefficients *D*_1_ and *D*_2_, respectively. Let *θ*^*j*^_*i*_ be the concentration of *X*_*i*_ particles in the region [*jh*, (*j* + 1)*h*) × [0, *L*] × [0, *L*], for some grid-spacing *h* > 0 and integer *j*, and let *p*^*j*^_*i*_ be the probability that a random point in [*jh*, (*j* + 1)*h*) × [0, *L*] × [0, *L*] can accommodate a single particle of species *X*_*i*_. Then we can approximately model diffusion of particles as a ‘hopping’ between neighbouring grid points. A particle of *X*_*i*_ can hop from [*jh*, (*j* + 1)*h*) × [0, *L*] × [0, *L*] to [(*j* + 1)*h*, (*j* + 2)*h*) × [0, *L*] × [0, *L*] with rate (*D*_*i*_/*h*^2^)*p*^*j*+1^_*i*_. Incorporating *p*^*j*^_*i*_ into the hopping rate accounts for the probability that a particle is blocked by crowders. Taking a mean-field approach to this description leads to a spatially discrete diffusion equation for the concentration of *X*_*i*_:2.1
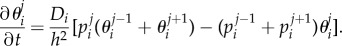
Similar mean-field equations have been derived for equal-sized particles, such as in [27,28]. The equations for *θ*^*j*^_1_ and *θ*^*j*^_2_ are not independent, but are rather coupled via the quantity *p*^*j*^_*i*_ which is naturally a function of both *θ*^*j*^_1_ and *θ*^*j*^_2_. The quantity *p*^*j*^_*i*_ is the probability that a random point in [*jh*, (*j* + 1)*h*) × [0, *L*] × [0, *L*] can accommodate a particle of *X*_*i*_, which is approximately given by scaled particle theory (SPT) [1,29]:2.2
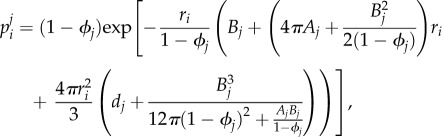
where 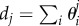, 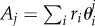, 
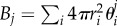
 and 
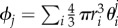
. Note that we are using the SPT formula for *p*^*j*^_*i*_ rather than the more usual 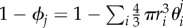 [28]. This is because we require *p*^*j*^_*i*_ to be the probability that a random point is surrounded by a sufficiently large empty region to at least accomodate a whole particle of radius *r*_*i*_. This probability is given by SPT, whereas 1 − *ϕ*_*j*_ is merely the probability that a random point can accommodate a point-particle, and is therefore an overestimate of the required quantity (for more information, see [30]).

Defining *θ*_*i*_(*x*) = *θ*^*j*=⌊*x*/*h*⌋^_*i*_ and *p*_*i*_(*θ*_1_(*x*), *θ*_2_(*x*)) = *p*^*j*=⌊*x*/*h*⌋^_*i*_, and taking the limit 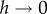, we use equation (2.1) to obtain continuous PDEs for the concentrations *θ*_*i*_:2.3
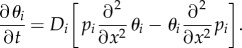
Again the PDEs for *θ*_1_ and *θ*_2_ are coupled via the functions *p*_*i*_. We now consider the case where *θ*_2_(*x*, *t*) ≪ *θ*_1_(*x*, *t*) for all *x* and *t*. It follows that *p*_*i*_(*θ*_1_, *θ*_2_) ≈ *p*_*i*_(*θ*_1_, 0). Intuitively, this means that the *X*_2_ concentration is so low that it does not affect the diffusion of any particles, but the *X*_1_ concentration affects the diffusion of both species. We therefore simply write *p*_*i*_(*θ*_1_). It follows that the diffusion equation for *X*_1_ is completely self-contained, while the diffusion equation for *X*_2_ depends on *X*_1_. We can write the two equations as2.4
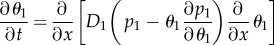
and2.5
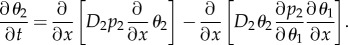
In other words, *X*_1_ obeys a nonlinear diffusion equation with diffusion coefficient *D*_1_(*p*_1_ − *θ*_1_(∂*p*_1_/∂*θ*_1_)), while *X*_2_ obeys a nonlinear advection–diffusion equation with diffusion coefficient *D*_2_*p*_2_ and velocity *D*_2_(∂*p*_2_/∂*θ*_1_)(∂*θ*_1_/∂*x*) in the positive *x*-direction. For more details on deriving nonlinear PDEs from lattice models, see [30,31].

We now further consider the case where *r*_2_ ≪ *r*_1_. Combining this with the earlier assumption that *θ*_2_(*x*, *t*) ≪ *θ*_1_(*x*, *t*), it follows that we are now considering a single small particle of type *X*_2_ diffusing amongst several large particles of type *X*_1_. Let *ε* = *r*_2_/*r*_1_. Perturbatively expanding equation (2.2) in small *ε* gives the following:2.6
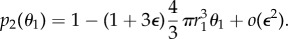
Furthermore, from the Stokes–Einstein relation, we have that *D*_1_/*D*_2_ = *ε*. It follows that the time scale on which *θ*_1_ changes is much slower than that of *θ*_2_. We can, therefore, make a quasi-stationarity assumption about *θ*_1_ on the time scale of *θ*_2_: we say *θ*_1_ = *θ*_1_(*x*). Note that this stationarity is consistent with our earlier biological observations that heterogeneous crowder distributions are maintained over long time scales. Finally, letting 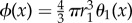 be the proportion of volume occupied by *X*_1_ at *x*, and writing *θ*(*x*, *t*) = *θ*_2_(*x*, *t*) and *D* = *D*_2_, we have a linear advection–diffusion equation for *X*_2_:2.7
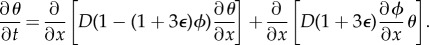
We, therefore, have a rigorously derived advection–diffusion equation for the concentration of small molecules diffusing in a completely generic crowder distribution *ϕ*(*x*). This PDE shows that particle motion is affected in two distinct ways. (i) The particle's local diffusion coefficient is rescaled by a factor of 1 − (1 + 3*ε*)*ϕ*(*x*), where *ε* is the ratio of small-to-large particle radii and *ϕ*(*x*) is the local proportion of volume occupied by crowders. This recovers the classical 1 − *ϕ* scaling in the case of point-particle diffusion (*ε* = 0). (ii) The particle moves with a velocity −*D*(1 + 3*ε*)(∂*ϕ*/∂*x*) in the positive *x*-direction, that is, a velocity directed towards less crowded regions and proportional to the gradient of the crowder distribution. If *ϕ* is constant (i.e. a uniform crowder density), this velocity becomes zero, and the particle will obey a standard diffusion equation (albeit with a reduced diffusion coefficient). Particle motion will generally be governed by the interplay between effects (i) and (ii), since particles will tend to move towards more dilute regions of space but will tend to move faster in those regions.

## 3. Applications

Using equation (2.7), we can investigate the motion of small molecules in a variety of crowder distributions. Of particular interest are the mean and variance (mean squared displacement, MSD) of *θ* as function of time. In particular, whether the variance is superlinear or sublinear, which would correspond to super- and subdiffusion, respectively.

The mean and variance of *θ* cannot be obtained directly from equation (2.7), so instead we write the solution of the advection–diffusion equation as a Taylor series in time:3.1
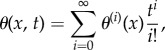
where *θ*^(*i*)^(*x*) = ∂^*i*^*θ*/∂*t*^*i*^|_*t*=0_. The time derivatives can be immediately obtained from equation (2.7) by thinking of the right-hand side as a differential operator acting on *θ*:3.2
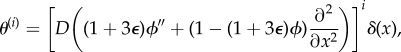
where we have assumed *θ*(*x*, 0) = *δ*(*x*), and *ϕ*′′ denotes the second derivative of *ϕ*(*x*). Each *θ*^(*i*)^ is then a sum of products of derivatives of *ϕ*(*x*) and *δ*(*x*).

The *n*th moment of *θ* is defined as3.3
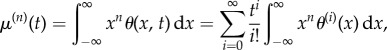
so that the variance is given by *μ*^(2)^(*t*) − (*μ*^(1)^(*t*))^2^. At very short times, the *t* term of the variance will dominate, so the particle motion will be diffusive. We can then investigate the transition to subsequent anomalous diffusion at short times by looking at the *t*^2^ term of the variance. If the coefficient of this term is positive, then the variance will be initially superlinear, and so the motion will be initially superdiffusive. Similarly, if the *t*^2^ term is negative, the motion will be initially subdiffusive. A zero coefficient for the *t*^2^ term denotes normal diffusion.

By squaring the expression for *μ*^(1)^(*t*), and observing that 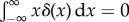, we find the coefficient of the *t*^2^ term in the expansion of the variance:3.4
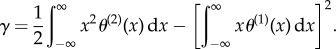
The initial anomalous diffusion follows immediately:3.5
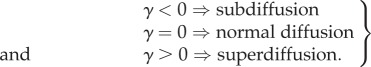


We now apply the advection–diffusion equation to a variety of physically plausible heterogeneous crowder distributions. Since our PDE is (i) derived from a lattice description, (ii) uses a mean-field assumption and (iii) uses the approximate SPT theory, it is not clear how accurate its predictions will be. We, therefore, also compare our PDE with hardsphere BD simulations, which suffer from none of these limitations.

**Figure 2.**
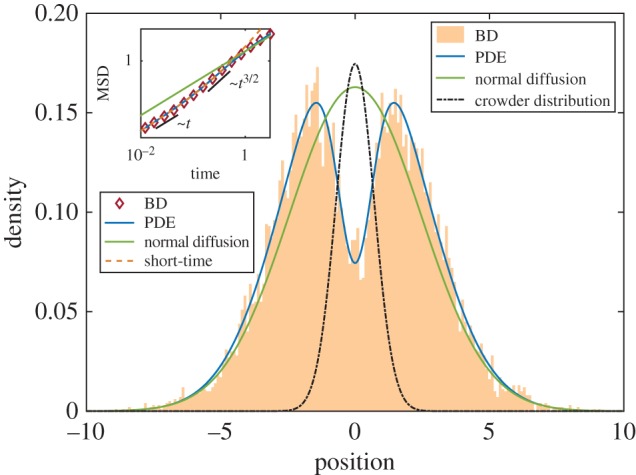
Inset: Mean-squared displacement against time for a small particle diffusing in a Gaussian distribution of crowders *ϕ*(*x*) = *k*e^−*x*^2^^. Main: Probability density of small particle location for the same system at time *t* = 3. Crowder distribution not to scale. Parameter values: *k* = 0.52, *ε* = 0.1, *D* = 1, *L* = 1, Δ*t* = 10^−5^. BD averaged over 10^5^ simulations.

First, we study a Gaussian crowder distribution *ϕ*(*x*) = *k*e^−*x*^2^^, where *k* is the maximum volume occupied. (Note that *k* must be less than 0.74, the densest sphere packing.) This could represent a local distribution of ribosomes, which are known to assemble near individual strands of mRNA [5,32]. The symmetry of this example implies that the mean of *θ* is zero for all times, but the variance may vary. Using this *ϕ* in equation (3.4) gives *γ* = 10*D*^2^*k*(1 + 3*ε*)(1 − *k*(1 + 3*ε*)). Since *k* < 0.74 and *ε* is ‘small’, say *ε* ≤ 0.1, it follows that *k*(1 + 3*ε*) < 1, and hence *γ* > 0. Therefore, a small particle in a Gaussian crowder distribution will transition from diffusive to superdiffusive motion at short times. In figure 2, we confirm this with BD simulations using the Cichocki–Hinsen algorithm [33]. In the inset, we plot MSD against time, and it is clear that our analytical theory is correct initially, and our PDE is correct for all times shown. In the main figure, we plot a snapshot of the distribution at a fixed time, where the PDE and BD both exhibit bimodal behaviour, clearly distinct from normal diffusion. The bimodal distribution arises because the small particle is directed (by the advection term in equation (2.7)) down one or other of the slopes of the Gaussian distribution.

**Figure 3.**
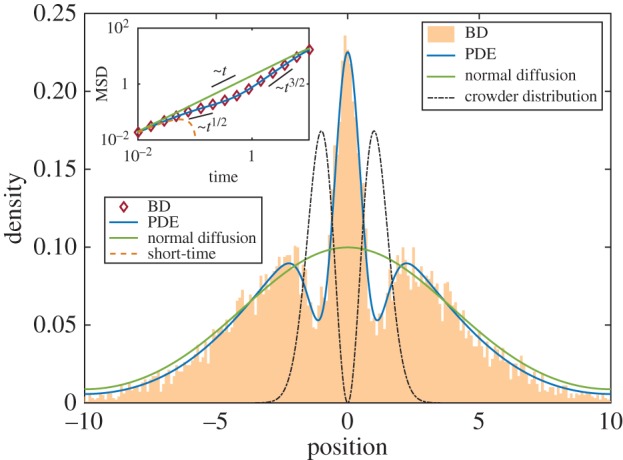
Inset: Mean-squared displacement against time for a small particle diffusing in a bimodal Gaussian distribution of crowders *ϕ*(*x*) = *kx*^2^e^1−*x*^2^^. Main: Probability density of small particle location for the same system at time *t* = 10. Crowder distribution not to scale. Parameter values: *k* = 0.52, *ε* = 0.1, *D* = 1, *L* = 1, Δ*t* = 10^−5^. BD averaged over 10^5^ simulations.

Next, we study a bimodal Gaussian crowder distribution *ϕ*(*x*) = *kx*^2^e^1−*x*^2^^, where again *k* is the maximum volume occupied. This could represent the bimodal distribution of macromolecules characteristic of cells undergoing division [22,23]. The symmetry of this example again implies that the mean of *θ* is zero for all times, but the variance may vary. Using this *ϕ* in equation (3.4) gives *γ* = − 10*D*^2^e*k*(1 + 3*ε*) < 0. Therefore, a small particle in a bimodal Gaussian crowder distribution will transition from diffusive to subdiffusive motion at short times. In figure 3, we confirm this with BD simulations. In the plot of MSD against time (inset), we observe that the particle motion transitions from diffusive to subdiffusive at short times, as predicted, but later becomes superdiffusive. In the main figure, we plot a snapshot of the distribution at a fixed time (*t* = 10), where the PDE and BD both exhibit trimodal behaviour, clearly distinct from normal diffusion. A number of effects give rise to this irregular behaviour: the small particle is initially trapped (by the advection term in equation (2.7)) between the two peaks of the bimodal crowder distribution—hence subdiffusion—but eventually, it will move past one of these peaks and be directed (by the advection term) down the outer slope—hence superdiffusion. At *t* = 10, for the parameter set chosen, there is a significant chance that the particle is still trapped in the central region, but also a significant chance that the particle has moved past one or other of the peaks, hence the trimodal behaviour.

**Figure 4.**
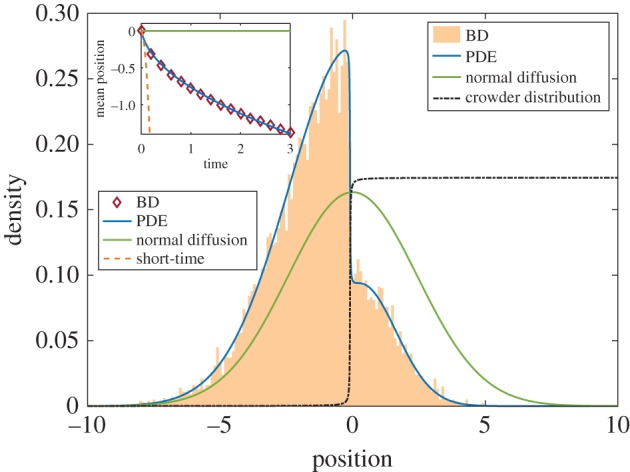
Inset: Mean particle position against time for a small particle diffusing in a step-like distribution of crowders *ϕ*(*x*) = (*k*/*π*)(arctan(*s*(*x* + *w*)) + *π*/2). Main: Probability density of small particle location for the same system at time *t* = 3. Crowder distribution not to scale. Parameter values: *s* = 100,  *w* = 0.1,  *k* = 0.52, *ε* = 0.1, *D* = 1, *L* = 1, Δ*t* = 10^−5^. BD averaged over 10^5^ simulations.

Finally, we study a step-like crowder distribution *ϕ* = (*k*/*π*) (arctan(*s*(*x* + *w*)) + *π*/2), where again *k* is the maximum volume occupied, *s* is a measure of the sharpness of the step and *w* is the distance between the step and the initial particle. This could represent a phase boundary such as the point where nucleoid meets cytosol. This example is asymmetric, so we expect the mean particle position to change with time, as well as the particle variance. We find that, at short times, the mean particle position is given by *μ*^(1)^(*t*) = − (2*Dks*(1 + 3*ε*)/*π*(1 + *s*^2^*w*^2^))*t* + *o*(*t*^2^), so that the particle performs directed motion towards the left (less crowded half) of the space. We also find that *γ* = (2/*π*^2^)*D*^2^*k*^2^*s*^2^(1 + 3*ε*)^2^ > 0, so the motion will initially transition from diffusive to superdiffusive. In figure 4, we confirm the change in mean position with BD simulations (inset). It is clear that our analytical theory is qualitatively correct, and our PDE is correct for all times shown. In the main figure, we plot a snapshot of the distribution at a fixed time, where the PDE and BD both exhibit non-Gaussian asymmetric behaviour, clearly distinct from normal diffusion. The steep slope of the crowder distribution causes the particle to be directed to the left (by the advection term in equation (2.7)) with high speed: this causes the particle to outrun normal diffusion in the negative half of the space. There is a small chance that the particle will diffuse into the right half of the space, but the diffusion coefficient here is significantly reduced so that normal diffusion is considerably faster.

## 4. Conclusion

In this article, we have shown that heterogeneous macromolecular crowding can lead to highly irregular motion of small particles, that differs wildly from the usual diffusion equation. From a microscopic model, we rigorously derived a simple advection–diffusion equation, equation (2.7), to describe the motion of a small particle in an arbitrary distribution of large crowder molecules, which agrees excellently with detailed BD simulations. The shape of the crowder distribution *ϕ*(*x*) can induce surprising small particle behaviour, such as bimodal and trimodal distributions, and directed motion. We also observed superdiffusive or subdiffusive motion, and both are possible in physically plausible crowder distributions. We further developed a fast analytical method to check whether a given crowder distribution *ϕ*(*x*) leads to super- or subdiffusive motion initially, and whether that motion is directed. This allows us to accurately predict the initial effect of any crowder distribution without solving the PDE.

There are two main consequences of our results. Firstly, they show that it is essential to incorporate subcellular heterogeneity into models of macromolecular crowding. The motion of a particle in a heterogeneous environment differs so greatly from its homogeneous counterpart, that ignoring heterogeneity could lead to erroneous modelling predictions. Secondly, our results suggest that cells might take advantage of heterogeneous crowding to direct particles towards or away from specific locations. For example, our results predict that a newly translated protein will diffuse quickly away from its parent mRNA molecule owing to the locally high concentration of ribosomes, thereby reducing the time taken to reach its destination.

It is worth noting that the work in this article ignores hydrodynamic interactions between particles, which are induced by the flow field in the surrounding fluid as a particle diffuses [34]. Such interactions are believed to be important to accurate modelling of *in vivo* diffusion [35], but are frequently ignored in simulations owing to their huge computational cost [12], which is due to the separation-dependent correlation between each particle's incremental Gaussian displacements at each time step [36]. By contrast, in lattice-based descriptions (and the Cichocki–Hinsen algorithm) it is assumed that only one particle moves at a time, and the direction of motion is independent of the other particles' motion (though not of their position). It is currently not possible to incorporate hydrodynamic effects into our model, though we are working on a way to do this which will hopefully be the subject of a future paper. However, we can make an educated guess about the likely impact. Batchelor showed that, in the diffusion of a single species of sphere, hydrodynamic effects tend to reduce the magnitude of steric effects, but not enough to offset them entirely [34]. We therefore expect hydrodynamic interactions to maintain the directed motion and altered diffusion coefficients, but with a reduced magnitude.

Nevertheless, while a more detailed model of crowding would incorporate many different crowder sizes, non-spherical particles and hydrodynamic interactions, the work in this article shows that a relatively simple steric model of crowding can lead to a wide variety of anomalous behaviours if crowder heterogeneity is taken into account.

## Supplementary Material

Matlab Code for Brownian dynamics

## Data accessibility

Matlab code for simulating hard-sphere Brownian dynamics is given in the electronic supplementary material.

## Appendix A. Brownian dynamics simulations

The BD algorithm used to produce the figures in this article is the Cichocki–Hinsen algorithm [33,37] with reflective boundaries in the *y*- and *z*-directions. The algorithm, with time-step Δ*t*, can be summarized as follows:

(1) Place small particle at 0. Place large particles in a non-overlapping configuration.
(2) Propose a new position for the small particle, at a Normal(0, 2*D*Δ*t*) increment from its current position. If the proposed position overlaps another particle or the boundary of the volume, reject it, otherwise accept it.
(3) For each large particle, in turn, propose a new position at a Normal(0, 2*εD*Δ*t*) increment from its current position. If the proposed position overlaps another particle or the boundary of the volume, reject it, otherwise accept it.
(4) Advance time by Δ*t*.
(5) Repeat steps 2–4 until sufficient time has elapsed.

We note that the large particles move with diffusion coefficient *Dε*, with *ε* ≪ 1, so that the large particle density is then essentially stationary on the time scale of interest.

Matlab code to perform the above algorithm is given in the electronic supplementary material.
